# The effect of social anxiety on threat acquisition and extinction: a systematic review and meta-analysis

**DOI:** 10.7717/peerj.17262

**Published:** 2024-05-09

**Authors:** Shannon Wake, Nicholas Hedger, Carien M. van Reekum, Helen Dodd

**Affiliations:** 1 School of Psychology and Clinical Language Sciences, University of Reading, Reading, United Kingdom; 2 University of Exeter, Exeter, United Kingdom

**Keywords:** Social anxiety, Threat acquisition, Threat extinction, Exposure therapy

## Abstract

Although exposure-based therapy has been found to be effective at alleviating symptoms of social anxiety disorder, it often does not lead to full remission, and relapse after treatment is common. Exposure therapy is based on theoretical principles of extinction of conditioned fear responses. However, there are inconsistencies in findings across experiments that have investigated the effect of social anxiety on threat conditioning and extinction processes. This systematic review and meta-analysis aimed to examine whether elevated levels of social anxiety are associated with abnormalities in threat conditioning and extinction processes. A second aim was to examine the sensitivity of various study designs and characteristics to detect social anxiety-related differences in threat conditioning and extinction. A systematic search was conducted, which identified twenty-three experiments for inclusion in the review. The findings did not demonstrate compelling evidence that high levels of social anxiety are associated with atypical threat conditioning or extinction. Further, when systematically examining the data, there was no convincing support that the use of a particular psychophysiological measure, subjective rating, or experimental parameter yields more consistent associations between social anxiety and conditioning processes during threat acquisition or extinction. Meta-analyses demonstrated that during threat extinction, the use of anxiety ratings as a dependent variable, socially relevant unconditioned stimuli, and a higher reinforcement schedule produced more detectable effects of social anxiety on compromised extinction processes compared to any other dependent variable (subjective or physiological) or experimental parameter. Overall, the results of this study suggest that social anxiety is not reliably related to deficits in conditioning and extinction processes in the context of laboratory-based Pavlovian conditioning paradigms.

## Introduction

Social anxiety disorder (SAD) is characterised by marked fear and avoidance of social or performance situations (DSM-V; American Psychiatric Association, 2013), due to the belief that negative evaluation will result in humiliation and rejection by others (Clark & Wells, 1995)^1^. The disorder has an estimated lifetime prevalence of 12.1% (Ruscio et al., 2008), higher than any other anxiety disorder, except specific phobia (Kessler et al., 2005). Social anxiety can lead to significant disability in education, employment, and relationships (Stein & Kean, 2000; Stein & Kean, 2000) and is associated with a variety of co-occurring conditions, including depression, generalised anxiety disorder, and substance abuse (Beesdo et al., 2007; Spence & Rapee, 2016).

Cognitive behaviour therapy (CBT) is the first-line intervention for the treatment of social anxiety (Pilling et al., 2013) and typically incorporates exposure therapy as a central component (Chesham, Malouff & Schutte, 2018; Heimberg, 2002). Exposure therapy is based on the theoretical principle of extinction of conditioned fear responses and involves gradual, repeated exposure to feared objects and contexts in the absence of the feared outcome (Craske et al., 2014). During extinction 1Portions of the text throughout the manuscript were previously published within a doctoral thesis written by Dr. Shannon Wake (Wake, 2021).learning, a conditioned stimulus (CS) is repeatedly presented in the absence of the feared outcome until fear of the CS gradually declines. Although exposure therapy has been found to be effective in alleviating symptoms of SAD (Jorstad-Stein & Heimberg, 2009; Ponniah & Hollon, 2008), it often does not lead to full remission, and relapse after treatment is common (Hofmann & Smits, 2008). Further, SAD is associated with poorer treatment outcomes compared with other anxiety disorders (Ginsburg et al., 2011; Hudson et al., 2015; Kerns et al., 2013), however, the reason for this is unclear (Evans, DM & Leigh, 2021). As threat extinction provides the (neuro)behavioural basis for exposure therapy (Craske et al., 2008), examining the relationship between social anxiety and threat conditioning and extinction may have implications for the treatment of SAD.

Most often, research on threat conditioning and extinction has employed a Pavlovian differential threat acquisition and extinction paradigm. During the acquisition phase, a neutral conditioned stimulus (CS+) is paired with an aversive unconditioned stimulus (US) so that the CS+ acquires the capacity to generate a defensive response when presented alone (conditioned response, CR). Another conditioned stimulus (CS-) serves as the comparison condition and hence is never paired with the US. Differential threat conditioning is typically observed by an increase in self-reported (*i.e.,* US expectancy) or physiological fear-related responses (*e.g.*, skin conductance response) to the CS+ compared to the CS-. During the extinction phase, the CS+ and CS- are both repeatedly presented in the absence of a US, leading to a decrease in the CR, as the CS+ loses its predictive value concerning the US. Extinction does not erase the learned threat association. Instead, it involves new safety learning which inhibits the expression of the original threat memory (Bouton, 1993; Milad & Quirk, 2012). Threat conditioning processes are considered significant in the pathogenesis and maintenance of pathological anxiety (Lonsdorf et al., 2017), the development and treatment of which can be modelled experimentally using threat acquisition and extinction paradigms.

In a meta-analysis that examined differences in classical threat conditioning and extinction of threat between 963 patients with stress and anxiety disorders and 1,222 healthy control subjects, Duits et al. (2015) indicated that during threat acquisition, fear responses to safety cues (CS-) were elevated in patients with anxiety compared to non-anxious controls. In contrast, during extinction, patients revealed increased fear responses towards the CS+ compared to healthy controls. Further, patients tended to demonstrate persistent differentiation between the CS+ and CS- throughout the extinction phase, indicating delayed or reduced extinction in patients diagnosed with anxiety disorders. However, Duits et al. (2015) did not differentiate between different subtypes of anxiety (*e.g.*, social anxiety, generalised anxiety, phobias *etc*.) except for PTSD (*i.e.,* PTSD *vs* ‘other anxiety groups’) in their review. The review also did not consider experiments that examined individuals with elevated levels of anxiety, but who might not have received a formal diagnosis of an anxiety disorder. In relation to social anxiety, previous models have suggested that social anxiety should be conceptualised as existing along a severity continuum, as many individuals experience severe symptoms without meeting the threshold for a clinical diagnosis of SAD (Bögels et al., 2010; Spence & Rapee, 2016). Therefore, such subclinical samples may provide valuable insight into social anxiety-linked threat acquisition and extinction processes that otherwise would be overlooked by excluding individuals with milder cases that fall beneath the diagnostic threshold (Chesham, Malouff & Schutte, 2018; Ruscio, 2010).

Social anxiety is particularly of interest in the context of the conditioning and extinction literature for several reasons. First, as discussed above, exposure treatment for social anxiety often does not lead to full remission, and relapse is common. Second, there are inconsistencies in findings across experiments that have investigated the effect of social anxiety on threat conditioning and extinction processes. Given that social anxiety is likely to be unique compared to other anxiety subtypes in terms of the amount of exposure to the feared stimulus that the individual experiences in their daily life, it could be suggested that individuals who have higher social anxiety may have particular difficulty learning to update threat associations to safety associations (Ruscio, 2010). It is almost impossible to avoid social situations entirely, but despite exposure, social anxiety is maintained. Therefore, the aim of the present study was to conduct a systematic review and meta-analyses of the literature on conditioning and extinction processes in relation to social anxiety to inform potential clinical avenues for exposure-based therapies that aim to promote the retention of the extinction memory.

An initial scoping review of research in this field indicated that there was considerable variation in task design, methodology and the dependent measures assessed across experiments. As such, we also examined the sensitivity of various study designs and characteristics, for example, the use of subjective *versus* psychophysiological fear measures, to detect social anxiety-related differences in threat conditioning and extinction. Within the broader threat conditioning literature, subjective report measures of fear learning encompass both cognitive (*i.e.,* ratings of CS-US expectancies or ‘risk’) and affective (*i.e.,* ratings of valence, arousal, fear/anxiety associated with a CS) components. Psychophysiological indices are commonly used alongside subjective report measures and have the advantage of not being subject to self-report biases (Lonsdorf et al., 2017). Psychophysiological indices also provide information about changes, of which we are often unaware, in different bodily response systems (*e.g.*, expressive responsivity or physiological arousal). The present systematic review and meta-analyses adopts a dimensional approach and includes research focused on clinical social anxiety as well as experiments that have investigated individual variation in social anxiety levels (*i.e.,* trait social anxiety).

## Method

### Literature search and information sources

The papers included in this review were identified *via* three separate literature searches and a previously published meta-analysis. The first literature search (Search 1) was conducted in July 2017 to identify articles that aimed to investigate the relationship between anxiety (across all anxiety subtypes) and threat acquisition and extinction. All studies identified as part of this search that focused on social anxiety were retained for the present review. Search 1 excluded studies that recruited patient samples because we were able to extract studies that had investigated the relationship between social anxiety disorder (SAD) and threat acquisition and extinction from the previously published meta-analysis carried out by Duits et al. (2015). We then conducted the second literature search (Search 2) in December 2019 to capture any additional relevant articles, including those focusing on either social anxiety disorder or trait levels of social anxiety, published between 2013 (after the end date of the article search for the Duits et al. (2015) meta-analysis) and December 2019^2^. 2Search 2 covered articles published between 2013–2019 as experiments that recruited patient populations (up until 2017) were excluded from Search 1 and therefore needed to be accounted for between 2013–2017 in Search 2.Further, to account for the passing of time since Search 2, a third literature search was conducted to identify any relevant articles that had been published between December 2019 and January 2022. By combining these approaches, we were able to capture all papers examining threat acquisition and extinction in relation to either social anxiety disorder or social anxiety conceptualised continuously published up to and including January 2022.

#### Search 1 (July 2017)

Search 1 was carried out in PsychInfo, Web of Science and PubMed and used the search terms (conditioned OR conditioning OR extinction) AND (anxi* OR phobi* OR worry OR panic), that had to be present in the title and/or abstract of the paper. After duplicates had been removed across databases, 6,038 records were identified, and abstracts were screened based on inclusion criteria. There were 456 full-text articles that were reviewed for eligibility. Of these, only six articles (Ahrens et al., 2014; Blechert et al., 2015; Ly & Roelofs, 2009; Olsson et al., 2013; Pejic et al., 2013; Tinoco-González et al., 2015) investigated trait social anxiety assessed with a self-report measure and these were hence selected for inclusion in the current systematic review. 

#### Extracting relevant studies from Duits et al. (2015) meta-analysis

The Duits et al. (2015) review examined experiments of threat conditioning and extinction in the anxiety disorders, including social anxiety disorder. Given that Search 1 excluded studies focusing on patient samples, we extracted articles from Duits et al. (2015) that recruited individuals with SAD. An additional three articles (Hermann et al., 2002; Lissek et al., 2008; Veit et al., 2002^3^) 3Veit et al. (2002) was later excluded from the current systematic review during the quality check (see “Risk of Bias in Individual Experiments” for details).were selected for inclusion in the current review on this basis.

#### Search 2 (December 2019)

Search 2 was carried out to identify experiments published between 2013 and December 2019 using the same databases as in Search 1. This date range was selected to gather articles published after the search end date of the Duits et al. (2015) review. The selection of studies was based on the following search terms that had to be present in the title and/or abstract of the paper: (conditioned OR conditioning OR extinction) AND social AND (anxi* OR phobi*). After removing duplicates between databases, 217 abstracts were extracted and screened. Thirteen full-text articles were then reviewed, of which five additional studies were included in the review (Reichenberger et al., 2017; Rabinak et al., 2017; Michalska et al., 2019; Ahrens et al., 2016; Shiban et al., 2015).

Reference lists of selected articles were screened, and one additional article was identified and included in the review (Schneider et al., 1999).

#### Search 3 (January 2022)

Search 3 was identical to Search 2 but search terms were set to capture articles that had been published between December 2019 and January 2022, to update the search. After removing duplicates between databases, 168 abstracts were extracted and screened. Thirteen full-text articles were then reviewed, of which seven additional were included in the review (Fung & Alden, 2020; Fyer et al., 2020; Reichenberger, Pfaller & Mühlberger, 2020; Savage et al., 2020; Stegmann et al., 2020; Wake et al., 2021a; Wake, Van Reekum & Dodd, 2021b).

**Figure 1:**
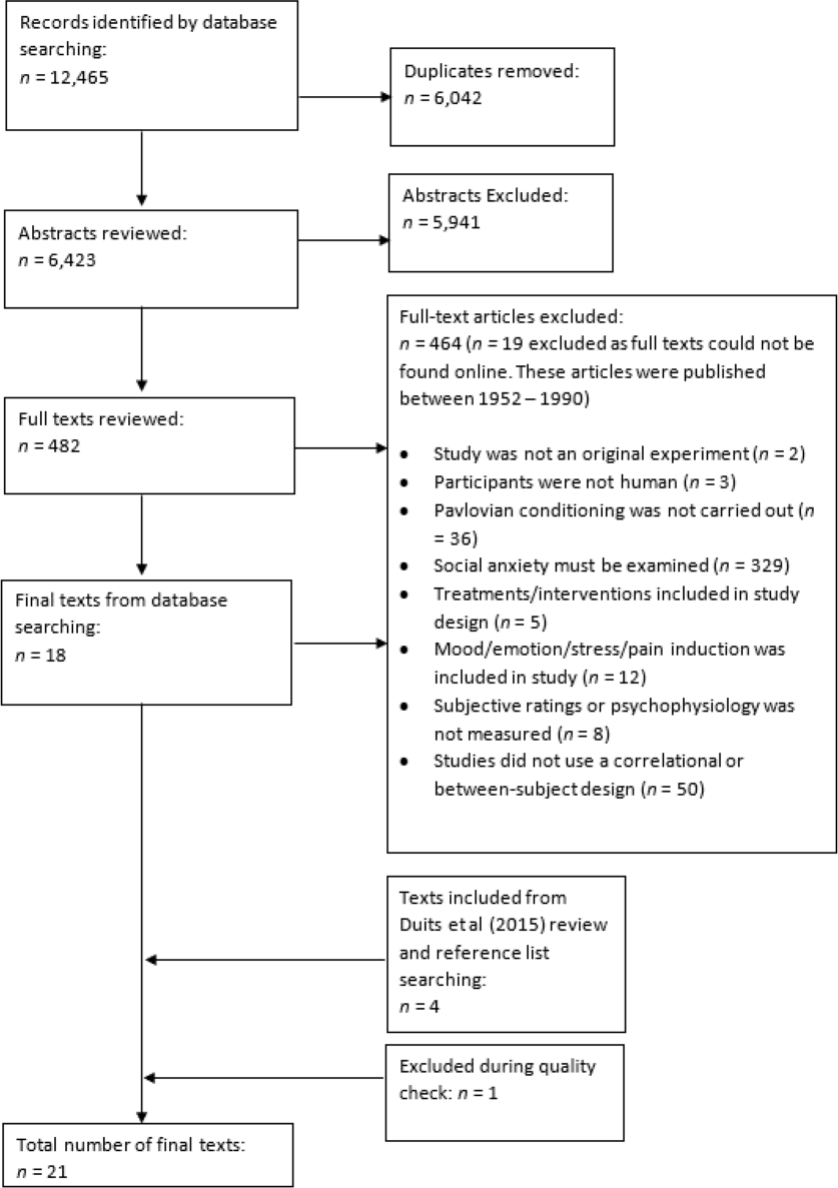
PRISMA Diagram. PRISMA Diagram combining search results across Search 1, Search 2, Search 3, and the extracted articles from the Duits et al. (2015) meta-analysis.

Across all searches, twenty-one articles were identified as relevant for inclusion in the current systematic review (see Fig. 1).

### Inclusion and exclusion criteria/eligibility criteria

Abstracts and full-text articles were only included in the review if they met the following eligibility criteria: (1) the study must be written in English, (2) the study must be an original, peer-reviewed experiment (*i.e.,* not a dissertation, case study or review article), (3) participants must be human, (4) classical aversive conditioning and extinction must be examined, (5) the study must recruit patients diagnosed with social anxiety disorder according to past or current DSM criteria (Search 2 and Search 3) or social anxiety must be measured using a standardised self-report measure of social anxiety (Search 1, Search 2, and Search 3), (6) the study must not incorporate a treatment or intervention, including medication and/or therapy, (7) there must not be a mood/emotion/stress/pain induction or manipulation, including training, at any point during the study, (8) Subjective (*i.e.,* ratings of valence/anxiety/expectancy) and/or psychophysiological (*i.e.,* startle/skin conductance/corrugator EMG/heart rate/pupil) outcome measurements must be used to index the conditioned fear response, and (9) the experiments must use a correlational or between-subjects design.

Outcome measures relating to brain imaging (*i.e.,* functional magnetic resonance imaging/positron emission tomography) were considered to be beyond the scope of the current review.

### Study selection

Study selection adhered to PRISMA guidelines (Liberati et al., 2009) (see Fig. 1). First, abstracts from all sources were screened against eligibility criteria (outlined above), followed by the full texts. The eligibility criteria for Search 1, Search 2, and Search 3 were identical, except for criterion 5 (see above). During the study selection of Search 1, articles were excluded if patients with a formal diagnosis of SAD had been recruited. Articles from Search 2 and Search 3 were not excluded based on the recruitment of patient populations. An article could be excluded at any stage of the screening process based on a ‘no’ response to any of the eligibility criteria; the first criterion that was not met was recorded as the reason for the rejection. Where criteria were coded as unclear (in the absence of any definite ’no’ codes) at the abstract stage, articles were put through to the full-text screening.

### Piloting and inter-rater reliability

Eligibility criteria and search terms were piloted before Search 1 was conducted, and eligibility criteria were altered accordingly (criterion 6 and 7 were added). After completion of piloting, two coders (the first author, SW, and a postgraduate student, IH) checked the first 2,000 abstracts of Search 1 (33%). Based on these 2,000 abstracts, a high level of inter-rater reliability was found between coders for accept/reject decisions (*K* = .95, *p* < .001). The remaining abstracts were coded by SW.

To ensure the reliability of the criteria for the full-text screening, SW and a second postgraduate student (MA) both assessed the first 249 full-texts (55%) against the eligibility criteria. Again, there was good inter-rater reliability (*k* = .69, *p* < .001) between coders for accept/reject decisions on the basis of these 249 full texts. SW coded the remaining articles.

Any disagreements between coders at either stage of the screening were discussed to reach a consensus.

SW coded all abstracts and full texts for Search 2 and Search 3 given that reliability had already been established in the context of Search 1.

### Risk of bias in individual experiments

The Standard Quality Assessment Criteria for Evaluating Primary Research Papers was used to indicate the overall quality of all the included studies (Kmet, Cook & Lee, 2004) (Table S1). Studies were scored on fourteen items on a checklist that assessed study design, methodology, accurate reporting of results, and conclusions made. Depending on the extent to which they met the criteria (2 = yes, 1 = partly, 0 = not addressed), a summary score was calculated for each study. Kmet, Cook & Lee (2004) suggest that a relatively conservative cut off point for article inclusion is 75%. One of the selected papers (Veit et al., 2002) had a score of 64% and therefore, based on this criterion, was excluded from the current review, leaving a total of twenty-one research articles. Otherwise, the studies included were of high quality, with scores ranging from 77%–95%. Only articles that fulfilled the criteria of the risk of bias assessment are referred to in the remainder of the review (*i.e.,* n = twenty-one published articles).

### Data extraction

Once all of the full texts had been screened, the first author extracted all of the relevant data, including (1) sample characteristics, (2) methodology, (3) statistical analyses conducted, and (4) the studies outcome measures that were relevant to the aims of the review. The above information was used to conduct a qualitative systematic review of the twenty-three included experiments in relation to the aims of the study.

To calculate the effect sizes included in the meta-analyses, the first author extracted the means and standard deviations of responses towards the CS+ and the CS- separately for each group of participants (*i.e.,* individuals with higher social anxiety and individuals with lower social anxiety). Where available, means and standard deviations were extracted from the text, tables, or figures (using WebPlotDigitizer software; Drevon, Fursa & Malcolm (2017)) included in the individual papers.

Means and standard deviations were extracted from fourteen of the twenty-three experiments during the acquisition phase and seven of the twenty-three experiments during the extinction phase. We were unable to extract the required information to calculate effect sizes from the remaining studies, either because the means and standard deviations of the effects of interest were not reported, or because the other reported statistics (F, p or t values) did not allow a straightforward conversion to an effect size due to complex, or unclearly reported model specifications, or lacked descriptive statistics to interpret the direction of the effect. As such, fourteen experiments were included in meta-analyses of acquisition effects and seven experiments are included in meta-analyses of extinction effects (See Table 1).

**Table 1:** Study characteristics and experimental parameters.

Author	Analyses conducted	No. of trials (Each Phase)		No. CS	Type CS	Type US	Reinforcement rate	Duration CS	ITI length
		ACQ	EXT						
Ahrens et al. (2014)	Systematic review & Meta-analyses (Acq; Ext)	20	20	3	Faces	Critical vocal statements	100%	5,000 ms	4,500–5,500 ms
Ahrens et al. (2016)	Systematic review & Meta-analyses (Acq)	12	0	2	Faces	Female scream	75%	6,000 ms	9,000–12,000 ms
Blechert et al. (2015)	Systematic review& Meta-analyses (Acq; Ext)	4	8	3	Faces	Critical vocal statement	100%	6,000 ms	25–45 s
Fung & Alden (2020)	Systematic review	30	30	3	Faces	Critical vocal statement	100%	1,000 ms	NR
Fyer et al. (2020)	Systematic review & Meta-analyses (Acq; Ext)	12	20	2	Coloured lightbulbs in lamp	Electric Shock	67%	3,000 ms	NR
Hermann et al. (2002)	Systematic review & Meta-analyses (Acq; Ext)	60	25	2	Faces	Odour	100%	5,000 ms	17–21 s
Lissek et al. (2008)	Systematic review & Meta-analyses (Acq)	8	8	3	Faces	Critical vocal statement	100%	8,000 ms	2,000 ms
Ly & Roelofs (2009)	Systematic review	2 (2 x CS+) 4 (1 x CS-)	0	3	Faces	Critical vocal statement	100%	5,000 ms	25–45 s
Michalska et al. (2019)	Systematic review	10	8	2	Coloured cartoon bells	Loud alarm	80%	8,000 ms	8–21 s
Olsson et al. (2013)	Systematic review	15	15	2	Faces	Electric shock	100%	6,000 ms	12–15 s
Pejic et al. (2013)	Systematic review	16	26	2	Faces	Critical vocal statement	100%	8,000 ms	16.25–18.75 s
Rabinak et al. (2017)	Systematic review & Meta-analyses (Acq; Ext)	20	20	3	Outdoor scenes with coloured streetlights	White noise	60%	4,000 ms	4,000–9,000 ms
Reichenberger et al. (2019)	Systematic review	12	12	2	Social agents	Electric shock OR critical vocal statement	75%	8,000 ms	20 s
Reichenberger, Pfaller & Mühlberger (2020)	Systematic review & Meta-analyses (Acq & Ext)	32	48	2	Social agents	Sound of spitting, airblast and verbal rejection	75%	NR	NR
Savage et al. (2020)	Systematic review & Meta-analyses (Acq)	25	0	2	Coloured spheres	White noise	33%	2,000 ms	12,000 ms
Schneider et al. (1999)	Systematic review	30	10	2	Faces	Odour	100%	6,000 ms	4,000 ms
Shiban et al. (2015)	Systematic review	8	12	2	Social agents	Airblast and scream	75%	8,000 ms	20 s
Stegmann et al. (2020)	Systematic review	30	0	2	Faces	Fearful expression and female scream	80%	3,000 ms	2,000–2,500 ms
Tinoco-González et al. (2015), Exp 1	Systematic review & Meta-analyses (Acq)	8	4	3	Faces	Critical vocal statement	100%	8,000 ms	2,000 ms
Tinoco-González et al. (2015), Exp 2	Systematic review & Meta-analyses (Acq)	8	4	3	Faces	Critical vocal statement	100%	8,000 ms	2,000 ms
Wake et al. (2021a), Exp 1	Systematic review & Meta-analyses (Acq)	40	0	2	Faces	Electric shock and critical statement	50%	4,000 ms	6,000–8,000 ms
Wake et al. (2021a), Exp 2	Systematic review & Meta-analyses (Acq; Ext)	24	32	2	Faces	Electric shock and critical statement	50%	4,000 ms	6,000–8,000 ms
Wake, Van Reekum & Dodd (2021b)	Systematic review & Meta-analyses (Acq)	24	0	2	Faces	Electric shock and critical statement	50%	4,000 ms	6,000–8,000 ms

**Notes.**

ACQ Acquisition EXT Extinction CS Conditioned stimulus US Unconditioned stimulus ITI Inter-trial interval NR Not reported

The primary effect size index used to quantify effects during threat acquisition and extinction was Cohen’s d, *i.e.,* the difference between responses towards the CS+ *versus* the CS- between two groups (*i.e.,* individuals with higher social anxiety and individuals with lower social anxiety). Initially, a Cohen’s d was calculated for the within-subject difference between responses towards the CS+ and CS- (*i.e.,* for individuals with higher social anxiety and individuals with lower social anxiety separately) using the following Cohen’s d calculation for within-subject designs that uses the mean difference as the numerator average and standard deviation as the denominator (Cumming, 2012; Lakens, 2013). The overall effect size (formula X) was thus calculated by subtracting the Cohen’s d for the difference between responses towards the CS+ *versus* the CS- for individuals with lower social anxiety from the Cohen’s d for the difference between responses towards the CS+ *versus* the CS- for individuals with higher social anxiety. A positive overall value of Cohen’s *d* represents an increased conditioned response for individuals with higher social anxiety relative to lower social anxiety.

\begin{eqnarray*}d= \frac{{M}_{CSdiffHA}}{ \frac{S{D}_{1}+S{D}_{2}}{2} } - \frac{{M}_{CSdiffLA}}{ \frac{S{D}_{1}+S{D}_{2}}{2} } \end{eqnarray*}

Formula X

### Meta-analyses model

Effect size outcomes were modelled with a random effects model due to its tolerance of heterogeneous effect sizes and conservative nature of estimation (Schmidt, Oh & Hayes, 2009). Heterogeneity across effect sizes was measured by Cochran’s Q statistic. Unless reported otherwise, parameter estimates were obtained *via* restricted maximum likelihood estimation, owing to its superior accuracy given a smaller number of studies (López-López et al., 2014).

Many samples contributing to the analysis produced multiple effects, entailing a nested structure. We modelled this by incorporating a hierarchical model specification that nested effects within their corresponding samples, thereby estimating random effects at both the effect and sample level. We thus used the following random effects structure ‘∼1 — sample_id/effect_id’. All models were fit using the ‘rma.uv’ function in the R package ‘metafor’ (Viechtbauer, 2010).

### Moderator analyses

To model heterogeneity in effect sizes, we proceeded in two ways. We first fit single-moderator models to examine the effect of each moderator in isolation. This phase allowed us to determine a subset of potentially explanatory variables for further exploration. Statistical tests of model coefficients were computed Wald-type chi squared statistics.

To test multiple combinations of moderators, we employed automated model selection and fit all combinations of models up to and including two-way interactions. These models were compared and ranked the basis of Akaike’s information criterion (AIC). This information-theoretic approach (Burnham & Anderson, 2002) allowed us to determine the ‘importance’ of each coefficient on the basis of their summed Akaike weights across the population of models. This approach, implemented in the ‘MuMin’ R package (Bartoń, 2020), depends on repeated evaluations of a ‘full model’ formula that includes all moderators. If such a model could not be specified on account of being underdetermined (too many coefficients), we used the single moderator phase to remove the most uninformative coefficients based on their *p* value.

**Table 2:** Sample characteristics.

Authors	Definition of social anxiety: Trait/Diagnosis	No. of participants and *M* (Age)						Study location
		Whole sample		Controls		Socially anxious		
			No. Female		No. Female		No. Female	
Ahrens et al. (2014)	Trait	NA	NA	23 (21.6)	14	24 (20.1)	15	Germany
Ahrens et al. (2016)	Diagnosis	NA	NA	26 (26.46)	8	29 (27.66)	12	Germany
Blechert et al. (2015)	Trait	NA	NA	38 (20.1)	20	33 (20.3)	23	USA
Fung & Alden (2020)	Trait	263 (20.67)	200	NA	NA	NA	NA	Canada
Fyer et al. (2020)	Diagnosis	NA	NA	64 (28.0)	34	41 (29.5)	22	USA
Hermann et al. (2002)	Diagnosis	NA	NA	19 (27.2)	0	14 (31.1)	0	Germany
Lissek et al. (2008)	Diagnosis	NA	NA	18 (28.11)	11	20 (30.63)	11	USA
Ly & Roelofs (2009)	Trait	NA	NA	22 (21.55)	6	26 (19.42)	18	The Netherlands
Michalska et al. (2019)	Trait	59 (13.35)	NR	NA	NA	NA	NA	USA
Olsson et al. (2013)	Trait	43 (23)	21	13 (NR)	NR	14 (NR)	NR	USA
Pejic et al. (2013)	Trait	41 (23.49)	23	NR	NR	NR	NR	Germany
Rabinak et al. (2017)	Diagnosis	NA	NA	15 (22.87)	11	16 (27.00)	14	USA
Reichenberger et al. (2019)	Trait	44 (21.53)	32	NA	NA	NA	NA	Germany
Reichenberger, Pfaller & Mühlberger (2020)	Trait	NA	NA	27 (NR)	14	26 (NR)	14	Germany
Savage et al. (2020)	Diagnosis	NA	NA	60 (20.3)	35	41 (19.85)	24	Australia
Schneider et al. (1999)	Diagnosis	NA	NA	12 (NR)	0	12 (NR)	0	Germany
Shiban et al. (2015)	Trait	40 (22)	37	NR	NR	NR	NR	Germany
Stegmann et al. (2020)	Trait	67 (24.1)	48	NA	NA	NA	NA	Germany
Tinoco-González et al. (2015), Exp 1	Diagnosis	NA	NA	16 (30.06)	13	16 (25.81)	13	Spain
Tinoco-González et al. (2015), Exp 2	Trait	NA	NA	20 (21.55)	15	20 (20.15)	15	Spain
Wake et al. (2021a), Exp 1	Trait	83 (19.61)	83	NA	NA	NA	NA	UK
Wake et al. (2021a), Exp 2	Trait	92 (19.66)	92	NA	NA	NA	NA	UK
Wake, Van Reekum & Dodd (2021b)	Trait	80 (20.1)	80	NA	NA	NA	NA	UK

**Notes.**

NA Not applicable NR Not Reported

## Results

### Sample characteristics

The twenty-one articles identified and included in this review were published between 1999 and 2022. The majority of experiments (*n* = 9) contained German samples, while the remaining experiments included samples from the USA (*n* = 6), the UK (*n* = 3), Australia (*n* = 1), Canada (*n* = 1), Spain (*n* = 1) and The Netherlands (*n* = 1) (see Table 2). Fourteen experiments used a self-report measure to quantify levels of social anxiety, including the Liebowitz Social anxiety scale (*n* = 2; Blechert et al., 2015; Tinoco-González et al., 2015), a pre-screening questionnaire based on DSM-IV criteria (*n* = 1; Ahrens et al., 2014), the Brief Fear of Negative Evaluation Scale (*n* = 1; Ly & Roelofs, 2009), a maternal report of Social Reticence (*n* = 1; Michalska et al., 2019), the Social Interaction Anxiety Scale (*n* = 1; Fung & Alden, 2020), the German version of the Social Phobia and Anxiety Inventory (*n* = 1; Stegmann et al., 2020), the Rejection Sensitivity Questionnaire (*n* = 1; Olsson et al., 2013), and the Social Phobia Inventory (*n* = 6; Pejic et al., 2013; Reichenberger et al., 2017; Reichenberger, Pfaller & Mühlberger, 2020; Shiban et al., 2015; Wake et al., 2021a; Wake, Van Reekum & Dodd, 2021b). The remaining experiments (*n* = 8) included samples of adults diagnosed with SAD and healthy control participants (see Table 2). Tinoco-González et al. (2015) reported two separate experiments within their paper, one of which recruited a clinical sample of patients with SAD and the other measured social anxiety using the Liebowitz Social Anxiety Scale. Wake et al. (2021a) also included two separate experiments in their paper. Only one experiment was included from all other articles, so a total of twenty-three separate experiments from twenty-one research articles are referred to in this review. All experiments, apart from Michalska et al. (2019), recruited adult samples.

### Systematic review

Please see Tables 1, 2, and 3 for specific details of the sample characteristics, outcome measures and experimental parameters employed across the twenty-three experiments.

**Table 3:** Dependent variables. A check mark (✓) indicates that the dependent variable was collected in the experiment.

Authors	Psychophysiology				Subjective ratings				
	SCR	HR	Corrugator	FPS	US expectancy	Fear/Anxiety	Arousal	Valence	
Ahrens et al. (2014)							✓	✓	
Ahrens et al. (2016)	✓	✓			✓		✓	✓	
Fung & Alden (2020)					✓	✓		
Fyer et al. (2020)	✓							
Blechert et al. (2015)								✓	
Hermann et al. (2002)	✓	✓	✓	✓	✓		✓	✓	
Lissek et al. (2008)				✓		✓	✓	✓	
Ly & Roelofs (2009)	✓				✓			
Michalska et al. (2019)	✓					✓		
Olsson et al. (2013)	✓							
Pejic et al. (2013)	✓					✓	✓	✓	
Rabinak et al. (2017)					✓			
Reichenberger et al. (2019)	✓			✓	✓	✓		
Reichenberger, Pfaller & Mühlberger (2020)						✓		
Savage et al. (2020)	✓						✓	✓	
Schneider et al. (1999)								✓	
Shiban et al. (2015)		✓		✓				✓	
Stegmann et al. (2020)					✓		✓	✓	
Tinoco-González et al. (2015), Exp 1				✓		✓	✓	✓	
Tinoco-González et al. (2015), Exp 2				✓		✓	✓	✓	
Wake et al. (2021a), Exp 1	✓				✓	✓		
Wake et al. (2021a), Exp 2	✓				✓	✓		
Wake, Van Reekum & Dodd (2021b)	✓				✓			

**Notes.**

SCR Skin Conductance Response HR Heart Rate FPS Fear-Potentiated Startle US Unconditioned Stimulus

#### Clinical social anxiety

Eight experiments compared patients with SAD to healthy control participants (HCs) to examine the effect of SAD on threat conditioning and extinction processes. Seven of these experiments explored the impact of SAD during acquisition and, of these, five experiments also explored the relationship between SAD and threat extinction. One study that recruited clinical samples did not carry out separate analyses to explore the impact of SAD on the acquisition and extinction phases, but examined the data across phases (see Table 1).

Within studies focused on SAD, the number of dependent variables (DV) measured when examining threat acquisition and extinction varied across studies (range one to seven dependent variables, see Table 3). For acquisition, three out of seven experiments found an effect of social anxiety on differential conditioning on at least one dependent variable (DV) (Hermann et al., 2002, 4/8 DVs; Lissek et al., 2008, 1/4 DVs; Rabinak et al., 2017, 1/1 DVs); none found effects consistently across multiple dependent variables. Similarly, three out of five experiments that examined the impact of SAD during the threat extinction phase found an effect of SAD on at least one dependent variable (Fyer et al., 2020, 1/1; Hermann et al., 2002, 2/8 DVs; Tinoco-González et al., 2015, Exp 1, $ \frac{1}{4} $ DVs). Further, Schneider et al. (1999) did not find an effect of SAD on differential conditioning in valence ratings (0/1 DVs) when investigating the relationship between SAD and conditioning processes across both phases.

#### Trait social anxiety

Fifteen experiments studied the relationship between trait social anxiety and threat conditioning and extinction. Fourteen of these experiments investigated the effect of social anxiety during threat acquisition and ten of these experiments also examined threat extinction. One study that assessed the relationship between levels of social reticence and threat conditioning processes collapsed the data across the acquisition and extinction phase in the analysis of dependent variables (see Table 1).

Studies focused on trait social anxiety included a range in the number of dependent variables measured (range 1 to 4 DVs). Seven out of thirteen experiments found a significant effect of trait social anxiety on differential conditioning for at least one dependent variable during threat acquisition (Fung & Alden, 2020; Ly & Roelofs, 2009, 1/ $ \frac{21}{2} $ DVs; Olsson et al., 2013, 1/1 DVs; Pejic et al., 2013, 2/4 DVs; Reichenberger, Pfaller & Mühlberger, 2020, 1/1; Shiban et al., 2015, 1/3 DVs; Tinoco-González et al., 2015 Exp 2, 1/ $ \frac{41}{4} $ DVs); none found effects consistently across dependent variables. During the threat extinction phase, four out of ten experiments found an effect of trait social anxiety on at least one dependent variable (Ahrens et al., 2014, 2/2 DVs; Blechert et al., 2015, 1/1 DVs; Olsson et al., 2013, 1/1 DVs; Shiban et al., 2015, 1/3 DVs); Ahrens et al. (2014) found effects consistently across two dependent variables. One study that examined the effect of trait social anxiety on dependent variables across the entire paradigm (Michalska et al., 2019, 0/2 DVs), did not find an effect of trait social anxiety during on conditioning processes.

### Dependent variables

### Psychophysiology

#### Skin conductance response.

Twelve out of twenty-three experiments recorded skin conductance response (SCR) (see Table 3). Ten experiments measured SCR during threat acquisition, of which two (Ly & Roelofs, 2009; Olsson et al., 2013) reported an effect of social anxiety on SCR towards the CS+ and CS- during acquisition. Eight studies found no significant relationship between social anxiety and SCR during acquisition. Ly & Roelofs (2009) reported that high socially anxious participants demonstrated marginally higher SCR during the first CS- trial of acquisition. Olsson et al. (2013) reported that high socially anxious individuals displayed a larger differential conditioned response towards the CS+ compared to low socially anxious participants.

Six out of twenty-three experiments measured SCR during threat extinction of which three reported effects of social anxiety on SCR towards the CS. Three studies did not find effects of social anxiety on SCR towards the CS. Fyer et al. (2020) reported that individuals with SAD demonstrated an increase in SCR towards the CS+ during late extinction leading to significant CS+ *vs* CS- differences. However, the mean CS+/CS- SCR differences of the SAD group (Early and Late Extinction) did not differ significantly from healthy controls. Hermann et al. (2002) reported that control subjects demonstrated differential SCR between the CS+ and CS- only during the first half of the extinction phase, while patients with SAD maintained differential SCR throughout the entire extinction phase. Olsson et al. (2013) demonstrated that high socially anxious individuals did not reveal successful extinction of the conditioned response in SCR across extinction when the CS was angry faces.

Michalska et al. (2019) examined the relationship between social anxiety and SCR towards CS collapsed across the entire threat conditioning and extinction paradigm and did not report an effect of social anxiety on SCR within their experiment.

#### Fear-potentiated startle.

Six experiments measured fear-potentiated startle (FPS) during threat acquisition (see Table 3). One of these experiments reported an effect of social anxiety on FPS during threat acquisition (Lissek et al., 2008), in that socially anxious subjects demonstrated a greater startle response for the CS paired with a socially relevant negative (*i.e.,* negative statements) US *versus* CSs paired with a neutral (*i.e.,* neutral statements) and positive (*i.e.,* positive statements) US respectively, but no effect of CS type on FPS was found among healthy control subjects. Five studies found no significant relationship between social anxiety and FPS during threat acquisition.

All six of the experiments that recorded FPS during threat acquisition also examined the effect of social anxiety on FPS during threat extinction. None of these experiments reported an effect of social anxiety on FPS towards the CS+ and CS- during threat extinction.

#### Heart rate.

Three experiments measured heart rate during threat acquisition and two experiments measured heart rate during extinction (see Tables 1 and 3). None of these experiments reported an effect of social anxiety on heart rate during CS+ and CS- trials during either the acquisition or extinction phase.

#### Corrugator EMG response.

One study measured corrugator EMG response towards the CS+ and CS- during threat acquisition and extinction (see Table 3). In this study there was an effect of social anxiety on the corrugator response elicited by the CS during threat acquisition, in that healthy control subjects, but not patients with SAD, showed an ‘at trend’ differential conditioning response of the right, but not left, corrugator muscle, but not during threat extinction (Hermann et al., 2002). There was no effect of social anxiety in corrugator response during threat extinction.

### Subjective ratings

#### Valence ratings

Eleven experiments measured self-reported valence ratings towards the CS during acquisition (see Table 3). Nine studies found no significant relationship, while two of these experiments reported a relationship between levels of social anxiety and valence ratings (Pejic et al., 2013; Shiban et al., 2015). Pejic et al. (2013) reported that higher social anxiety was associated with an increase in ratings of unpleasantness towards the CS after threat acquisition, relative to lower levels of social anxiety. Further, Shiban et al. (2015) found that individuals with low levels of social anxiety rated the CS+ as more unpleasant compared to the CS-. However, individuals with high levels of social anxiety did not demonstrate a difference in valence ratings towards the CS+ and CS-, suggesting generalisation of the conditioned response across CS during threat acquisition in this group.

Eight of the above experiments also recorded self-reported valence ratings towards the CS during threat extinction. Five of these studies found no significant relationship, while three of these experiments demonstrated effects of social anxiety on valence ratings to the CS+ and CS- during threat extinction (Ahrens et al., 2014; Blechert et al., 2015; Shiban et al., 2015). Ahrens et al. (2014) found that after threat extinction, low socially anxious individuals no longer discriminated between the three CS (CS+ negative, CS- neutral, CS- positive) in valence ratings, however, high socially anxious subjects continued to rate the CS+ negative as more unpleasant compared to the CS- neutral and CS- positive. Blechert et al. (2015) reported that high socially anxious subjects rated the CS+ negative as more unpleasant compared to low socially anxious subjects. However, group differences for the CS- neutral and CS- positive did not reach significance. Further, Shiban et al. (2015) found that low socially anxious individuals rated the CS+ as more unpleasant compared to the CS-. In contrast, high socially anxious individuals did not demonstrate discrimination between the CS+ and CS- in valence ratings.

Schneider et al. (1999) investigated the effect of social anxiety on valence ratings towards the CS+ and CS- across threat acquisition and extinction and found that patients with SAD rated the CS+ as more negative compared to HC subjects.

#### US expectancy

Nine experiments measured US expectancy towards the CS after acquisition (see Table 3). Seven of these studies found no significant relationship, while two experiments reported an effect of social anxiety (Hermann et al., 2002; Rabinak et al., 2017). Hermann et al. (2002) reported that in comparison to HC subjects, socially anxious patients gave overall higher US expectancy ratings, which was due to enhanced US expectancy towards the CS-. Rabinak et al. (2017) also found that patients with SAD showed greater US expectancy towards the CS- during early and late threat acquisition compared to HCs.

Five experiments recorded ratings of US expectancy during threat extinction (see Tables 1 and 3). Four of these experiments found no significant relationship, while one of these experiments reported an effect of social anxiety on US expectancy towards the CS (Hermann et al., 2002). Hermann et al. (2002) reported that simple contrasts comparing US expectancy ratings during early and late extinction (towards both CS) demonstrated that HCs, and not patients with SAD, reduced their US expectancy over time.

#### Arousal

Nine experiments measured self-reported arousal elicited by the CS during threat acquisition (see Table 3), Seven of these experiments found no significant relationship, while two experiments found a relationship between levels of social anxiety and arousal ratings (Hermann et al., 2002; Tinoco-González et al., 2015). Hermann et al. (2002) found that in comparison to HCs, patients with SAD rated the CS as overall more arousing, and this was accounted for by higher ratings of arousal towards the CS-. Further, Tinoco-González et al. (2015, Exp 2) reported that high trait socially anxious individuals rated the CS+ negative as more arousing than the CS- positive, however, there was not a difference in ratings of arousal towards the CS+ negative and CS- positive for low trait socially anxious individuals.

Six experiments measured self-reported levels of arousal towards the CS during threat extinction (see Tables 1 and 3). Four of these experiments found no significant relationship, while two of these experiments reported an impact of social anxiety on ratings of arousal (Hermann et al., 2002; Tinoco-González et al., 2015, Exp 1). Hermann et al. (2002) reported that patients with SAD gave higher arousal ratings, compared to HCs, and only differentiated between the CS+ and CS- during early extinction. Further, HC subjects rated the CS+ as more arousing compared to the CS-. Tinoco-González et al. (2015, Exp 1) reported that arousal ratings towards the CS- positive were higher for patients with SAD compared to HCs.

#### Fear/Anxiety ratings

Ten experiments measured levels of fear/anxiety elicited in response to the CS+ and CS- during threat acquisition (see Table 3). Seven of these experiments found no significant relationship, while three of these experiments reported a relationship between the level of social anxiety and ratings of fear elicited by the CS during acquisition. Fung & Alden (2020) reported that, for participants with social anxiety scores one standard deviation above the mean social anxiety score, subjective ratings of anxiety elicited by the CS+(hostile) increased, and subjective ratings of anxiety towards the CS-(neutral) decreased across acquisition. Whereas, for participants with social anxiety one standard deviation below the mean social anxiety score, subjective anxiety ratings towards both the CS+(hostile) and CS-(neutral) increased across acquisition. Pejic et al. (2013) reported that a higher degree of social anxiety was associated with a stronger increase in fear ratings towards the CS post threat acquisition. Reichenberger, Pfaller & Mühlberger (2020) reported that self-reported fear ratings demonstrated that successful differential threat conditioning took place during the acquisition phase, with the exception of men with increased social anxiety.

Eight experiments examined fear/anxiety ratings towards the CS during threat extinction. None of these experiments reported an effect of social anxiety on fear/anxiety ratings elicited by the CS during threat extinction.

### Experimental parameters

#### CS type

Socially relevant CSs, including facial expressions and ’social agents’ in virtual reality, were employed in eighteen experiments (see Table 1). During the acquisition phase, nine experiments found no significant relationship while nine experiments found an effect of social anxiety on differential conditioning (Fung & Alden, 2020; Hermann et al., 2002; Lissek et al., 2008; Ly & Roelofs, 2009; Olsson et al., 2013; Pejic et al., 2013; Reichenberger, Pfaller & Mühlberger, 2020; Shiban et al., 2015, Tinoco-González et al., 2015, Exp 2). During threat extinction, six experiments using socially relevant CSs found an effect of social anxiety on conditioning processes (Ahrens et al., 2014; Blechert et al., 2015; Hermann et al., 2002; Olsson et al., 2013; Shiban et al., 2015; Tinoco-González et al., 2015, Exp 1). Four experiments incorporated socially irrelevant CSs (coloured bells and coloured streetlights) (see Table 1). Rabinak et al. (2017) found an effect of social anxiety during the acquisition phase, but not the extinction phase. Fyer et al. (2020) found an effect of social anxiety during late extinction, but not across the whole extinction phase or during the acquisition phase. Michalska et al. (2019) and Savage et al. (2020) did not find an effect of social anxiety on conditioning processes during either acquisition or extinction.

#### US type

A socially relevant US, including vocal insults (*i.e.,* “you disgust me”) and critical statements (*i.e.,* “get lost”), were used in twelve experiments (See Table 1). Four experiments combined and administered both a critical statement and an electric shock as US. Six of these experiments found no significant relationship, while six experiments found an effect of social anxiety during acquisition (Fung & Alden, 2020; Lissek et al., 2008; Ly & Roelofs, 2009; Pejic et al., 2013; Reichenberger, Pfaller & Mühlberger, 2020; Tinoco-González et al., 2015 Exp 2). During threat extinction, one experiment found an effect of social anxiety on conditioning processes (Blechert et al., 2015) and two experiments found a marginal effect of social anxiety during threat extinction (Ahrens et al., 2014; Tinoco-González et al., 2015 Exp 1).

One study (Reichenberger et al., 2017) included US type as two between-subject conditions; one of which administered an electric shock and the other an air-blast to the neck with a spitting sound followed by the insult “get lost” over headphones. This study did not find that social anxiety was associated with compromised threat conditioning or extinction in either condition.

The remaining nine experiments did not use socially relevant US (*i.e.,* female scream, odour, loud alarm, electric shock, white noise, air blast) (see Table 1). Four of these experiments did not find a significant relationship, while five of experiments (Hermann et al., 2002; Olsson et al., 2013; Rabinak et al., 2017; Shiban et al., 2015) reported an effect of social anxiety on responses towards the CS during threat acquisition. During threat extinction, four experiments that did not employ socially relevant US found an effect of social anxiety on conditioning processes (Fyer et al., 2020; Hermann et al., 2002; Olsson et al., 2013; Shiban et al., 2015).

#### Reinforcement schedule

Eleven experiments used a 100% reinforcement schedule during the acquisition phase (see Table 1). Four of these studies found no significant relationship, while seven of these experiments (Fung & Alden, 2020; Hermann et al., 2002; Lissek et al., 2008; Ly & Roelofs, 2009; Olsson et al., 2013; Pejic et al., 2013; Tinoco-González et al., 2015, Exp 2) found a marginal effect of social anxiety on responses towards the CS in at least one dependent variable during threat acquisition. During threat extinction, six of the ten experiments that used a 100% reinforcement schedule (Ahrens et al., 2014; Blechert et al., 2015; Hermann et al., 2002; Olsson et al., 2013; Tinoco-González et al., 2015, Exp 1 and Exp 2) found an effect of social anxiety on conditioning processes.

Three of the experiments employed a 75% reinforcement schedule during threat acquisition (see Table 1). One of these experiments found an effect of social anxiety during acquisition (Reichenberger, Pfaller & Mühlberger, 2020), and another (Shiban et al., 2015) found an effect of social anxiety on conditioning processes during threat acquisition and extinction.

Two studies used an 80% reinforcement schedule (see Table 2), but did not find that social anxiety was associated with differences in threat conditioning across dependent variables during threat acquisition or extinction. Another study (Rabinak et al., 2017) employed a 60% reinforcement schedule and found an effect of social anxiety on conditioning processes during threat acquisition, but not during threat extinction. One study used a 67% reinforcement schedule (Fyer at al., 2020), and found an effect of social anxiety during late extinction. Three studies used a 50% reinforcement schedule (Wake et al., 2021a, Exp1; Wake et al., 2021a, Exp2; Wake, Van Reekum & Dodd, 2021b) and another study used a 33% reinforcement schedule (Savage et al., 2020), but did not report effects of social anxiety on acquisition or extinction.

### Meta-analyses

### Threat acquisition effects

Acquisition effects were taken from 14 studies. These studies contributed 14 samples, which comprised of 454 healthy controls and 399 participants with social anxiety. Ten of these samples contributed multiple effects, entailing 41 effects in total.

### Global outcomes.

**Figure 2:**
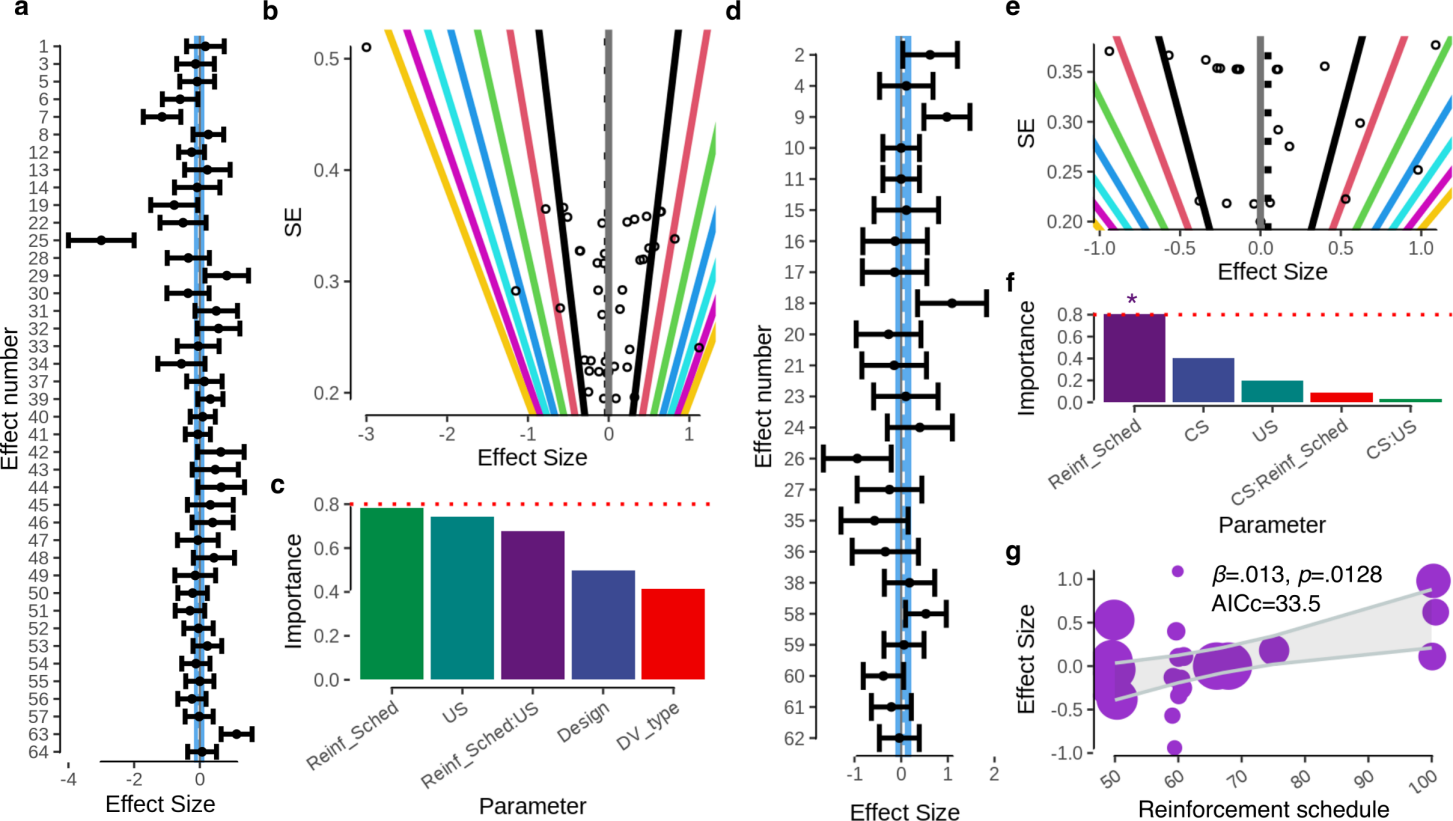
Meta-analyses. (A) Forest plot of effect sizes for threat acquisition effects. Shaded blue region is 95% confidence interval of the random effects model. (B) Funnel plot for acquisition effects, diagonal lines depict *p* values (outward from center: 0.05, 0.01, 0.001, *etc*.). (C) Moderator importances (summed Akaike weights) across the population of fitted models. The dashed red line indicates a cutoff of 0.8, which is sometimes used to delineate between informative and uninformative variables. Only the five most informative variables are shown. (D) Forest plot for threat extinction effects. (F) Moderator importances for extinction effects. The star indicates a moderator that has exceeded the 0.8 threshold. (G) Depicts the final model of extinction effects, as estimated by the model-selection procedure. Reinforcement schedule is plotted as the single moderator of effect size. The shaded grey region indicates the 95% confidence interval of the model prediction. Sizes of points are inversely proportional to their variance so as to emphasize more heavily weighted effects.

Figure 2A depicts a forest plot of the data and random-effects model outcomes. The random-effects model was not significant and estimated a very small, negative effect *d* = −.009 [−.212,.195], *p* = .934, AIC = 71.62. Expressed differently, the probability of superiority, *i.e.,* that a person with social anxiety picked at random would demonstrate an increased conditioned response compared to a person picked at random from the HC group, was estimated to be 50% (*i.e.,* chance level). The test for heterogeneity was significant Q(40) = 123.76, *p* < .001. According to Cook’s distances, only one strongly negative effect (which is clearly visible on the left of the forest plot, see Fig. 2A) was determined as being particularly influential (taken from Hermann et al., 2002). Further inspection revealed no obvious coding errors and so this study was retained in the analysis.

### Publication bias.

A funnel plot of effect sizes can be seen in Fig. 2B. Evidence of publication bias was limited: rank-sign test revealed no evidence of a relationship between sampling variances and effect magnitudes (T = .044, *p* = .696).

### Moderators.

No effect of experimental design was detected Q(1) = 1.2948, p = .255, there was no difference between studies that examined trait-level of social anxiety (*d* =  − 0.112, [−0.384, 0.160], p =.421) and clinical group designs (*d* = 0.13, [−0.185, 0.444], p =.419). No effect of DV was detected Q(9) = 5.89, *p* = 0.751. Of the DVs, only US expectancy elicited a detectable effect overall and this was in the negative direction (*d* =  − 0.593, [−1.176, −0.011], *p* = .046).

No effect of CS type was detected (Q(1) = 0.276, *p* = .599), there was no difference in effect magnitude between studies employing a social CS (*d* = 0.017, [−0.216, 0.251], p = .884) and non-social CS (*d* =  − 0.133, [−0.643, 0.376], *p* = .609). Similarly, no effect of US type was detected (Q(2) = 2.701, *p* = .259), there was no difference in effect magnitude between studies employing a social US (*d* = 0.090, [−0.201, 0.381], *p* = .546) and non-social US (*d* =  − 0.300, [−0.703, 0.103], *p* = .144) or both (*d* = .094, [−0.309, 0.496], *p* = .648). No effect of reinforcement schedule was detected Q(1) = 0.840, *β* = 0.004, [−0.005, 0.012], *p* = .359

### Moderator importance.

A ‘full model’ with all moderators could not be fit to the data. We therefore removed CS type from the analysis on the basis of its high *p* value. We additionally binarised the DV factor into a moderator that coded whether the DV was subjective or physiological to reduce the number of coefficients to be estimated.

The best model, according to AICc, included reinforcement schedule, US type and their interaction AICc(7) = 76.51. However, the model-averaged importance of each moderator value indicated none that exceeded 0.8, which is often used as a cutoff to differentiate between important and unimportant variables. Given this, and also considering that the second-ranking model included only the intercept, we opted to be conservative and retain this empty (no moderator) model as the final model (AICc(3) = 78.17). The model-averaged coefficients are shown in Table S2.

### Threat extinction effects

Threat extinction effects were taken from seven studies. These studies contributed seven samples, which comprised of 231 healthy control subjects and 193 participants with SAD. Five of these samples contributed multiple effects, entailing 23 effects in total.

### Global outcomes.

Figure 2D depicts a forest plot of the data and random-effects model outcomes. The random-effects model estimated a very small, positive effect *d* = 0.047 [−.131, .224], *p* = .607, AIC = 33.27. The probability of superiority, *i.e.,* that a person with social anxiety picked at random would demonstrate compromised threat extinction compared a healthy control subject, was estimated to be 51%. The test for heterogeneity was significant Q(22) =49.90, *p* < .001. Cook’s distances did not indicate any influential effects indicative of outliers.

### Publication bias.

A funnel plot of effect sizes can be seen in Fig. 2E. Evidence of publication bias was limited: rank-sign test revealed no evidence of a relationship between sampling variances and effect magnitudes (T =.044, *p* = .696).

### Moderators.

No effect of experimental design was detected Q(1) = 1.036, *p* = 0.309, there was no difference between studies that examined trait-levels of SA (*d* =  − 0.268, [−0.157, 0.692], *p* = 0.216) and clinical group designs (*d* =  − 0.140, [−0.352, 0.324], *p* = 0.935). An effect of DV was detected Q(8) = 18.724, *p* = 0.016. Of the DVs, anxiety ratings produced the largest detectable effects in the positive direction *d* = 0.987, [0.332, 1.642], *p* = 0.003. Holm-corrected *post-hoc* tests indicated significantly larger effects with anxiety ratings as the DV relative to US Expectancy t(14) =4.07, *p* = 0.041.

No effect of CS type was detected (Q(1) = 1.639, *p* = 0.201), there was no difference in effect magnitude between studies employing a social CS (*d* =  − 0.185, [−0.669, 0.298], *p* = 0.452) and non-social CS (*d* =  − 0.181, [−0.102, 0.464], *p* = 0.211). An effect of US type was detected (Q(2) = 6.648, *p* = 0.036). Holm-corrected *post-hoc* tests revealed that this was mostly driven by social US stimuli (Q(2) = 0.487, [0.116, 0.859], *p* = 0.010) having larger effect sizes than both non-social stimuli *β* = 0.558, t(20) = 2.538, *p* = 0.059 and combinations of both social and non-social stimuli *β* = 0.497, t(20) = 2.047, *p* = 0.108. An effect of reinforcement schedule was also detected, such that more reinforcement led to larger effects Q(1) = 0.129, *β* = 0.013, [0.003, 0.023], *p* = .0128

### Model selection and moderator importance.

A ‘full model’ with all moderators could not be fit to the data. We therefore removed CS type from the analysis on the basis of its high *p* value. The model-averaged importances of each moderator are depicted in Fig. 2F. The best model, according to AICc, included reinforcement schedule as the sole moderator AICc(4) = 33.506. This model is depicted in Fig. 2G. The model-averaged coefficients are shown in Table S3.

## Discussion

The purpose of this article was to synthesis the available research to examine whether elevated levels of social anxiety are associated with compromised threat conditioning and extinction processes. Overall, the findings across the systematic review and meta-analyses do not demonstrate compelling evidence that high levels of social anxiety are associated with atypical threat conditioning or extinction.

A second aim of this review was to investigate the sensitivity of various study designs and characteristics to detect social anxiety-related differences in threat conditioning and extinction. The included experiments were highly heterogeneous in their design, but there was little indication across findings that any particular design features or dependent variables were associated with more consistent effects of social anxiety. For example, across twenty-three experiments, there was no evidence that the experiments that had recruited patients diagnosed with SAD found more robust or consistent effects of social anxiety during threat acquisition and extinction compared to experiments that used self-report measures of trait social anxiety. Further, the systematic review demonstrated that there was no compelling support that the use of a particular psychophysiological measure (SCR, FPS, heart rate or corrugator response), subjective rating (valence, US expectancy, arousal, or fear/anxiety) or experimental parameter (CS type, US type or reinforcement schedule) yielded more consistent associations between social anxiety and threat conditioning or extinction processes compared to any other. Meta-analyses revealed that, under specific conditions (*i.e.,* the use of anxiety ratings as the dependent variable, a socially relevant US, and a higher reinforcement schedule), there was some evidence for social anxiety related difference in extinction processes.

Previous literature has argued that the experimental context might impact the expression of individual differences variables on outcome measures during threat conditioning and extinction studies (Lissek, Pine & Grillon, 2006; Lonsdorf et al., 2017). However, our findings do not support this suggestion in the case of social anxiety. Although the meta-analyses demonstrated that the use of anxiety ratings as a dependent variable, socially relevant US and higher reinforcement schedules were associated with effects of social anxiety during threat extinction, effect sizes were small and, when combined with evidence from the systematic review, unconvincing. For example, only two out of the eight experiments that recorded anxiety ratings during threat extinction were included in the meta-analyses (for reasons outlined in the Methods section above). As such, six relevant studies (all of which found no significant relationship between social anxiety and anxiety ratings during threat extinction) were not represented in the meta-analyses. Overall, if the effect of social anxiety on threat conditioning and extinction were robust, we would expect the effect to translate across study design parameters. Given that it does not, it is plausible to argue that social anxiety is not associated with atypical conditioning and extinction processes.

Associative learning is central to the development and maintenance of maladaptive fears (Beckers et al., 2023). For example, individuals with social anxiety fear innocuous social situations (conditioned stimuli) because they are associated with threatening outcomes (unconditioned stimuli). In the case of SAD, there is evidence to demonstrate that being bullied as a child can lead to social situations being associated with humiliation and rejection (Mineka & Zinberg, 2006). However, many individuals suffering from social anxiety do not recall direct traumatic conditioning experiences, suggesting that other important pathways contribute to the development and maintenance of SAD (Beckers et el., 2023). An explanation that aligns with the Clark & Wells (1997) model is that a contributory maintaining factor of social anxiety may be specific core beliefs and assumptions that inflate the threat level of social stimuli. Therefore, the findings of this review indicate that deficits in fear conditioning and extinction learning are unlikely to be a core issue in social anxiety and, instead, that cognitive processes such as negative biases, dysfunctional beliefs and attentional biases may be more relevant. Previous work has shown that patients with social anxiety interpret ambiguous social events as more negative and mildly negative social events as more catastrophic compared to other anxious patients or non-anxious control participants (Wells & Clark, 1997). Further, patients with social anxiety, compared to control participants, are more likely to remember negative interpersonal interactions (O’Banion & Arkowitz, 1977), evaluate their social behaviour negatively and make internal attributions for social failure (Girodo, Dotzenroth & Stein, 1981). Such evaluations of social situations may be particularly resistant to modification during exposure therapy because, in contrast to other fears and phobias, social cognitions are largely inaccessible to disconfirmation (Crask, 2003; Foa et al., 1996), in that individuals with social anxiety are reliant on estimates of what they believe others think of their behaviour. Given that we would not expect that exposure alone would challenge these cognitions, our results align with the idea that relapse after exposure treatment is explained not by deficits in safety learning but by the enduring negative cognitions that underpin social anxiety.

When interpreting the findings of the current review, an important consideration is that although some of the experimental work included in the systematic review and meta analyses employed socially relevant CS, it is possible that images of facial expressions and avatars within virtual reality are not capable of eliciting the fears or dysfunctional cognitions related to social anxiety (*i.e.,* the fear of negative evaluation or the allocation of attentional resources towards potential social threat). Although moderator analyses demonstrated that using a socially relevant US produced larger effect sizes during threat extinction, compared to both a non-social US, or the combination of social and non-social US, findings are inconsistent across studies. It appears likely that the social stimuli used in these studies may not be sufficiently ecologically valid to reliably uncover differences in threat conditioning associated with social anxiety. Therefore, future work should explore creative methods for eliciting levels of social threat comparable to social evaluation experienced in the real world to allow for the valid study of the relationship between social anxiety and threat conditioning and extinction processes.

Within this systematic review and associated meta-analyses there are a number of limitations that warrant discussion. First, only published experiments were included in the review. Although there was no convincing support for the aims outlined, it is likely that the magnitude of the association between social anxiety and threat conditioning and extinction would be further weakened with the inclusion of unpublished experiments, more likely to contain null results. Further, due to the considerable variation in experimental design and analyses across experiments and the lack of reporting of means and standard deviations of null findings in the majority of studies, we were not able to quantify effect sizes across all the relevant experiments for inclusion in the meta-analyses. A further limitation is that the search was not conducted in the traditional way because of a change in focus and passing of time during the research process. Nevertheless, we have provided an honest and clear account of how the searches were conducted and are confident that all relevant papers were identified through this process.

## Conclusion

This review indicates that there is relatively little support for the hypothesis that social anxiety is associated with compromised threat conditioning or extinction. There is significant heterogeneity across experiments in terms of design parameters and no evidence that results vary systematically across these parameters during threat acquisition. Meta-analyses indicated that there is some evidence to suggest that the use of anxiety ratings as a dependent variable, socially relevant US, and a higher reinforcement schedule are related to the detection of social anxiety related differences in threat extinction. However, overall, the results of this study suggest that social anxiety is not reliably related to deficits in conditioning and extinction processes in the context of laboratory-based Pavlovian conditioning paradigms.

### Registration and Protocol

This review was not registered and a protocol was not registered on PROSPERO.

## Supplemental Information

10.7717/peerj.17262/supp-1 Supplemental Information 1 PRISMA checklist

10.7717/peerj.17262/supp-2 Supplemental Information 2 Quality Assessment

10.7717/peerj.17262/supp-3 Supplemental Information 3 Model-Averaged Coefficients

10.7717/peerj.17262/supp-4 Supplemental Information 4 Rationale and Contribution

10.7717/peerj.17262/supp-5 Supplemental Information 5 Justification of additional author

## Additional Information and Declarations

### Data Availability

The following information was supplied regarding data availability: All data were extracted from the published articles included in the systematic review.
