# Supplementary material for: The effect of social anxiety on threat acquisition and extinction: a systematic review and meta-analysis
Source: PeerJ. 2024 May 9;12:e17262. doi: 10.7717/peerj.17262 (PMC11088819; doi:10.7717/peerj.17262)
Supplement: Supplemental Information 2 [file peerj-12-17262-s002.docx]

**Supplementary Materials**

Table 1 – Quality Assessment (Part 1)

|  | Question/objective sufficiently described? | Study design evident and appropriate? | Method of subject/comparison group selection or source of information/input variables described and appropriate? | Subject (and comparison group, if applicable) characteristics sufficiently described? | If interventional and random allocation was possible, was it described? | If interventional and blinding of investigators was possible, was it reported? | If interventional and blinding of subjects was possible, was it reported? |
| --- | --- | --- | --- | --- | --- | --- | --- |
| Ahrens et al. (2014) | 2 | 2 | 2 | 2 | N/A | N/A | N/A |
| Ahrens et al. (2016) | 2 | 2 | 2 | 2 | N/A | N/A | N/A |
| Blechert et al. (2015) | 2 | 2 | 1 | 2 | N/A | N/A | N/A |
| Fung et al. (2020) | 2 | 2 | 2 | 1 | N/A | N/A | N/A |
| Fyer et al. (2020) | 2 | 2 | 2 | 2 | N/A | N/A | N/A |
| Hermann et al. (2002) | 2 | 2 | 1 | 2 | N/A | N/A | N/A |
| Lissek et al. (2008) | 2 | 2 | 2 | 2 | N/A | N/A | N/A |
| Ly et al. (2009) | 2 | 2 | 2 | 2 | N/A | N/A | N/A |
| Michalska et al. (2018) | 2 | 2 | 2 | 2 | N/A | N/A | N/A |
| Olsson et al. (2013) | 2 | 2 | 2 | 2 | N/A | N/A | N/A |
| Pejic et al. (2013) | 2 | 1 | 2 | 1 | N/A | N/A | N/A |
| Rabinak et al. (2017) | 2 | 2 | 2 | 2 | N/A | N/A | N/A |
| Reichenberger et al. (2017) | 2 | 2 | 1 | 1 | N/A | N/A | N/A |
| Reichenberger et al. (2020) | 2 | 2 | 2 | 2 | N/A | N/A | N/A |
| Savage et al. (2020) | 2 | 2 | 2 | 2 | N/A | N/A | N/A |
| Schnieder et al. (1999) | 1 | 2 | 2 | 2 | N/A | N/A | N/A |
| Stegmann et al. (2020) | 1 | 2 | 2 | 2 | N/A | N/A | N/A |
| Shiban et al. (2015) | 2 | 2 | 2 | 1 | N/A | N/A | N/A |
| Tinoco-Gonzalez et al. (2014) | 2 | 2 | 2 | 2 | N/A | N/A | N/A |
| Veit et al. (2002) | 1 | 2 | 1 | 0 | N/A | N/A | N/A |
| Wake et al. (2021a) | 2 | 2 | 2 | 2 | N/A | N/A | N/A |
| Wake et al. (2021b) | 2 | 2 | 2 | 2 | N/A | N/A | N/A |

Quality Assessment (Part 2)

|  | Outcome and (if applicable) exposure measure (s) well defined? | Sample size appropriate? | Analytic methods described/justified and appropriate? | Some estimate of variance is reported for the main results? | Controlled for confounding? | Results reported in sufficient detail? | Conclusions supported by the results? | Total score/possible maximum score, % |
| --- | --- | --- | --- | --- | --- | --- | --- | --- |
| Ahrens et al. (2014) | 2 | 2 | 2 | 1 | 1 | 2 | 2 | 20/22 = 91% |
| Ahrens et al. (2016) | 2 | 2 | 2 | 2 | 1 | 2 | 2 | 21/22 = 95% |
| Blechert et al. (2015) | 1 | 2 | 2 | 1 | 2 | 2 | 2 | 19/22 = 86% |
| Fung et al. (2020) | 2 | 2 | 2 | 2 | 2 | 2 | 2 | 21/22 = 95% |
| Fyer et al. (2020) | 1 | 1 | 2 | 2 | 1 | 2 | 2 | 19/22 = 86% |
| Hermann et al. (2002) | 2 | 1 | 2 | 1 | 1 | 2 | 2 | 18/22 = 82% |
| Lissek et al. (2008) | 2 | 1 | 2 | 2 | 2 | 2 | 2 | 21/22 = 95% |
| Ly et al. (2009) | 2 | 2 | 2 | 1 | 1 | 2 | 2 | 20/22 = 91% |
| Michalska et al. (2018) | 1 | 2 | 2 | 1 | 1 | 2 | 2 | 19/22 = 86% |
| Olsson et al. (2013) | 1 | 1 | 1 | 1 | 1 | 2 | 2 | 17/22 = 77% |
| Pejic et al. (2013) | 2 | 2 | 1 | 1 | 1 | 2 | 2 | 17/22 = 77% |
| Rabinak et al. (2017) | 1 | 1 | 2 | 2 | 2 | 2 | 2 | 20/22 = 91% |
| Reichenberger et al. (2017) | 2 | 1 | 2 | 2 | 1 | 2 | 2 | 18/22 = 82% |
| Reichenberger et al. (2020) | 2 | 2 | 2 | 2 | 2 | 2 | 2 | 22/22 = 100% |
| Savage et al. (2020) | 2 | 2 | 2 | 2 | 2 | 2 | 2 | 22/22 = 100% |
| Schnieder et al. (1999) | 2 | 1 | 1 | 1 | 1 | 2 | 2 | 17/22 = 77% |
| Shiban et al. (2015) | 2 | 1 | 2 | 1 | 1 | 2 | 2 | 18/22 = 82% |
| Stegmann et al. (2020) | 1 | 1 | 2 | 2 | 1 | 2 | 2 | 18/22 = 82% |
| Tinoco-Gonzalez et al. (2014) | 2 | 1 | 2 | 1 | 2 | 2 | 2 | 20/22 = 91% |
| Veit et al. (2002) | 2 | 1 | 1 | 1 | 1 | 2 | 2 | 14/22 = 64% |
| Wake et al. (2021a) | 2 | 2 | 2 | 2 | 1 | 2 | 2 | 21/22 = 95% |
| Wake et al. (2021b) | 2 | 2 | 2 | 2 | 1 | 2 | 2 | 21/22 = 95% |

Note: Studies were scored on the extent to which they met the criteria (2 = yes fully addressed, 1 = partly addressed, 0 = not addressed). Items not relevant to a particular study designed were classified as “” and were subsequently excluded from the summary score. A summary score was then made for each study which resulted from scoring all applicable items on the scale and dividing by the total score possible for the applicable items. A full outline of the description of items and scoring guidelines can be found in Kmet and colleagues (2004) guidelines.
