# Supplementary material for: The effect of social anxiety on threat acquisition and extinction: a systematic review and meta-analysis
Source: PeerJ. 2024 May 9;12:e17262. doi: 10.7717/peerj.17262 (PMC11088819; doi:10.7717/peerj.17262)
Supplement: Supplemental Information 3 [file peerj-12-17262-s003.docx]

| **Supplemental Table 2.** Acquisition Phase: Model-Averaged Coefficients | | | | |
| --- | --- | --- | --- | --- |
| Moderator | Estimate | Std. Error | Z | *P* |
| Intercept | -1.589 | 1.596 | 1.000 | .319 |
| Reinforcement Schedule | 0.027 | 0.025 | 1.065 | .287 |
| Non-Social US | 1.943 | 1.783 | 1.090 | .276 |
| Social US | -0.952 | 1.279 | 0.744 | .457 |
| Reinforcement Schedule : Non-Social US | -0.037 | 0.029 | 1.279 | 0.201 |
| Trait Social Anxiety | 0.649 | 1.454 | 0.446 | .655 |
| Subjective Ratings | 0.320 | 0.847 | 0.378 | 0.705 |
| Subjective Ratings : Reinforcement Schedule | -0.007 | 0.017 | 0.429 | 0.668 |
| Subjective Ratings : Non-Social US | -0.076 | 0.231 | 0.331 | 0.741 |
| Subjective Rating : Social US | 0.409 | 0.940 | 0.435 | 0.663 |
| Trait Social Anxiety : Reinforcement Schedule | -0.006 | 0.015 | 0.425 | 0.671 |
| Trait Social Anxiety : Subjective Ratings | 0.033 | 0.162 | 0.202 | 0.840 |
| Trait Social Anxiety : Subjective Ratings : Reinforcement Schedule | 0.000 | 0.000 | 0.015 | 0.988 |

| **Supplemental Table 3.** Extinction Phase: Model-Averaged Coefficients | | | | |
| --- | --- | --- | --- | --- |
| Moderator | Estimate | Std. Error | Z | *p* |
| Intercept | -1.048 | 1.378 | 0.760 | 0.447 |
| Reinforcement Schedule | 0.016 | 0.021 | 0.763 | 0.445 |
| Social CS | 0.3853 | 1.2953 | 0.2975 | 0.7661 |
| Non-Social US | -0.0297 | 0.2618 | 0.1133 | 0.9098 |
| Social US | 0.0441 | 0.2489 | 0.1772 | 0.8594 |
| Social CS : Reinforcement Schedule | -0.0045 | 0.0192 | 0.2345 | 0.8146 |
| Reinforcement Schedule : Non-Social US | 0.0001 | 0.0040 | 0.0292 | 0.9767 |
| Arousal Ratings | 0.0000 | 0.0010 | 0.0012 | 0.9991 |
| US Expectancy Ratings | 0.0000 | 0.0017 | 0.0014 | 0.9989 |
| Valence Ratings | 0.0000 | 0.0007 | 0.0006 | 0.9995 |
| Fear Ratings | 0.0000 | 0.0009 | 0.0009 | 0.9993 |
| Fear Potentiated Startle | 0.0000 | 0.0011 | 0.0010 | 0.9992 |
| Skin Conductance Response | 0.0000 | 0.0009 | 0.0010 | 0.9992 |
| Unpleasantness Ratings | 0.0000 | 0.0009 | 0.0006 | 0.9995 |
| Anxiety Ratings | 0.0000 | 0.0009 | 0.0007 | 0.9994 |
| Social CS : US Expectancy | 0.0000 | 0.0000 | 0.0000 | 1.0000 |
| Arousal Ratings : Reinforcement Schedule | 0.0000 | 0.0000 | 0.0000 | 1.0000 |
| Valence Ratings : Reinforcement Schedule | 0.0000 | 0.0000 | 0.0000 | 1.0000 |
| Arousal Ratings : Non-Social US | 0.0000 | 0.0000 | 0.0000 | 1.0000 |
| Valence Ratings : Non-Social US | 0.0000 | 0.0000 | 0.0000 | 1.0000 |
