# Supplementary material for: The effect of social anxiety on threat acquisition and extinction: a systematic review and meta-analysis
Source: PeerJ. 2024 May 9;12:e17262. doi: 10.7717/peerj.17262 (PMC11088819; doi:10.7717/peerj.17262)
Supplement: Supplemental Information 4 [file peerj-12-17262-s004.docx]

**Supplementary Materials**

**Title: The Effect of Social Anxiety on Threat Acquisition and Extinction: A Systematic Review**

**Rationale – As stated in the Introduction section of the manuscript**

Cognitive behaviour therapy (CBT) is the first-line intervention for the treatment of social anxiety (Pilling et al., 2013) and typically incorporates exposure therapy as a central component (Chesham et al., 2018; Heimberg, 2002). Exposure therapy is based on the theoretical principle of extinction of conditioned fear responses and involves gradual, repeated exposure to feared objects and contexts in the absence of the feared outcome (Craske et al., 2014). During extinction learning, a conditioned stimulus (CS) is repeatedly presented in the absence of the feared outcome until fear of the CS gradually declines. Although exposure therapy has been found to be effective in alleviating symptoms of SAD (Jorstad-Stein & Heimberg, 2009; Ponniah & Hollon, 2008), it often does not lead to full remission, and relapse after treatment is common (Hofmann & Smits, 2008). Further, SAD is associated with poorer treatment outcomes compared with other anxiety disorders (Ginsburg et al., 2011; Hudson et al., 2015; Kerns et al., 2013), however, the reason for this is unclear (Evans et al., 2021). As threat extinction provides the (neuro)behavioural basis for exposure therapy (Craske et al., 2008), examining the relationship between social anxiety and threat conditioning and extinction may have implications for the treatment of SAD.

Most often, research on threat conditioning and extinction has employed a Pavlovian differential threat acquisition and extinction paradigm. During the acquisition phase, a neutral conditioned stimulus (CS+) is paired with an aversive unconditioned stimulus (US) so that the CS+ acquires the capacity to generate a defensive response when presented alone (conditioned response, CR). Another conditioned stimulus (CS-) serves as the comparison condition and hence is never paired with the US. Differential threat conditioning is typically observed by an increase in self-reported (i.e., US expectancy) or physiological fear-related responses (e.g., skin conductance response) to the CS+ compared to the CS-. During the extinction phase, the CS+ and CS- are both repeatedly presented in the absence of a US, leading to a decrease in the CR, as the CS+ loses its predictive value concerning the US. Extinction does not erase the learned threat association. Instead, it involves new safety learning which inhibits the expression of the original threat memory (Bouton, 1993; Milad & Quirk, 2012). Threat conditioning processes are considered significant in the pathogenesis and maintenance of pathological anxiety (Lonsdorf et al., 2017), the development and treatment of which can be modelled experimentally using threat acquisition and extinction paradigms.

In a meta-analysis that examined differences in classical threat conditioning and extinction of threat between 963 patients with stress and anxiety disorders and 1222 healthy control subjects, Duits et al. (2015) indicated that during threat acquisition, fear responses to safety cues (CS-) were elevated in patients with anxiety compared to non-anxious controls. In contrast, during extinction, patients revealed increased fear responses towards the CS+ compared to healthy controls. Further, patients tended to demonstrate persistent differentiation between the CS+ and CS- throughout the extinction phase, indicating delayed or reduced extinction in patients diagnosed with anxiety disorders. However, Duits et al. (2015) did not differentiate between different subtypes of anxiety (e.g., social anxiety, generalised anxiety, phobias etc.) except for PTSD (i.e., PTSD vs 'other anxiety groups') in their review. The review also did not consider experiments that examined individuals with elevated levels of anxiety, but who might not have received a formal diagnosis of an anxiety disorder. In relation to social anxiety, previous models have suggested that social anxiety should be conceptualised as existing along a severity continuum, as many individuals experience severe symptoms without meeting the threshold for a clinical diagnosis of SAD (Bögels et al., 2010; Spence and Rapee, 2016). Therefore, such subclinical samples may provide valuable insight into social anxiety-linked threat acquisition and extinction processes that otherwise would be overlooked by excluding individuals with milder cases that fall beneath the diagnostic threshold (Chesham et al., 2018; Ruscio, 2010).

Social anxiety is particularly of interest in the context of the conditioning and extinction literature for several reasons. First, as discussed above, exposure treatment for social anxiety often does not lead to full remission, and relapse is common. Second, there are inconsistencies in findings across experiments that have investigated the effect of social anxiety on threat conditioning and extinction processes. Given that social anxiety is likely to be unique compared to other anxiety subtypes in terms of the amount of exposure to the feared stimulus that the individual experiences in their daily life, it could be suggested that individuals who have higher social anxiety may have particular difficulty learning to update threat associations to safety associations (Ruscio, 2010). It is almost impossible to avoid social situations entirely, but despite exposure, social anxiety is maintained. Therefore, the aim of the present study was to systematically review the literature on conditioning and extinction processes in relation to social anxiety to inform potential clinical avenues for exposure-based therapies that aim to promote the retention of the extinction memory.

**Contribution - As stated in the Discussion section of the manuscript**

Overall, the findings do not demonstrate compelling evidence that high levels of social anxiety are associated with atypical threat conditioning or extinction. The included experiments were highly heterogeneous in their design, but there was little indication that any particular design features or dependent variables were associated with more consistent effects of social anxiety. For example, across the twenty-two experiments, there was no evidence that the experiments that had recruited patients diagnosed with SAD found more robust or consistent effects of social anxiety compared to experiments that used self-report measures of trait social anxiety. Further, there is no compelling support that the use of a particular psychophysiological measure (SCR, FPS, heart rate or corrugator response), subjective rating (valence, US expectancy, arousal, or fear/anxiety) or experimental parameter (i.e., CS type, US type and reinforcement schedule) yields more consistent associations between social anxiety and threat conditioning or extinction processes compared to any other. As such, it is clear that any effect that may exist is not large, robust or easily replicated. If the effect were robust, we would expect it to be observed more frequently across experiments and, where variation in results was found, for this to vary systematically according to study design parameters or outcome measures.

Despite the absence of compelling evidence for compromised safety learning in social anxiety, prior findings demonstrate that relapse after exposure therapy is common for individuals with anxiety disorders, including social anxiety (Craske et al., 2014; Graham & Milad, 2011; Hofmann and Smits, 2008). An explanation, that aligns with the Clark and Wells (1997) model, is that a contributory maintaining factor of social anxiety may be specific core beliefs and assumptions that inflate the threat level of social stimuli. Therefore, the findings of this review indicate that deficits in safety learning are unlikely to be a core issue in social anxiety and, instead, that cognitive processes such as negative biases, dysfunctional beliefs and attentional biases may be more relevant.

This review indicates that there is relatively little support for the hypothesis that social anxiety is associated with compromised threat acquisition or extinction. There is significant heterogeneity across experiments in terms of design parameters and no evidence that results vary systematically across these parameters. Future work in the laboratory should examine how compromised cognitive processes and avoidance behaviours, specifically related to social anxiety, affect the renewal of social fear after extinction learning to improve the efficacy of exposure-based treatments for social anxiety disorder.
