# Supplementary material for: The effect of social anxiety on threat acquisition and extinction: a systematic review and meta-analysis
Source: PeerJ. 2024 May 9;12:e17262. doi: 10.7717/peerj.17262 (PMC11088819; doi:10.7717/peerj.17262)
Supplement: Supplemental Information 5 [file peerj-12-17262-s005.docx]

21^st^ March 2024

Dear Professor Mark Boyes,

I am writing to outline the reasons for the addition of Dr Nicholas Hedger as an author on our paper, “The effect of social anxiety on threat acquisition and extinction: A systematic review and meta-analyses”. As recommended by yourself and Reviewer #1 in a previous review of the paper, we have updated the previously submitted manuscript with a meta-analyses of the data. To allow us to do this, we were required to recruit the expertise of Dr Nicholas Hedger which has been vital for the design and analysis of the meta-analyses.

Specifically, Dr Nicholas Hedger has contributed the following to the manuscript:

- Design of meta-analyses
- Conducted the meta-analyses
- Write up of meta-analyses within the results section, i.e., critical manuscript revision
- Produced Figure 2
- Interpretation of results related to meta-analyses
- Edits and approval of the final manuscript

I have commented within the revised manuscript to highlight sections that Dr Nocholas Hedger has contributed to the manuscript. Further, I can confirm that myself, Professor Carien van Reekum, and Professor Helen Dodd all approve the addition of Dr Nicholos Hedger as an author on the paper.

Yours sincerely,

Dr Shannon Wake

On behalf of all authors
